# Alleviation of Cognitive and Physical Fatigue with Enzymatic Porcine Placenta Hydrolysate Intake through Reducing Oxidative Stress and Inflammation in Intensely Exercised Rats

**DOI:** 10.3390/biology11121739

**Published:** 2022-11-29

**Authors:** Min Ju Kim, Ting Zhang, Keun Nam Kim, Gun Won Bae, Sun Myung Yoon, Yu Yue, Xuangao Wu, Sunmin Park

**Affiliations:** 1 Department of R&D, Unimed Pharmaceuticals Inc., Seoul 05567, Republic of Korea; 2 Department of Bio-Convergence System, Obesity/Diabetes Center, Hoseo University, Asan 31499, Republic of Korea; 3 Department of Food and Nutrition, Institute of Basic Science, Hoseo University, Asan 31499, Republic of Korea

**Keywords:** porcine placenta enzyme hydrolysates, exercise-induced fatigue, lactate, hypothalamus–pituitary–adrenaline axis, cognitive fatigue

## Abstract

**Simple Summary:**

The porcine placenta has been traditionally used as a potential treatment for liver damage, and enzymatic porcine placenta hydrolysate (EPPH) may promote efficacy. However, there are few studies of EPPH to demonstrate exercise-induced physical and cognitive fatigue. Daily intake of EPPH rich in glycine, glutamate, arginine, alanine, leucine, lysine, and oligopeptides (0.16–0.31 mL/kg bw; 5 mg nitrogen/mL) alleviates exercise-induced fatigue in an animal model by decreasing fatigue-related metabolite concentrations, including lactate, blood urinary nitrogen, and creatinine, reducing liver damage, and increasing glycogen deposition in the liver and skeletal muscles. EPPH inhibits stress-related signaling and elevates hippocampal neurotrophic factors to improve cognitive function. EPPH also reduces oxidative stress and inflammation in the skeletal muscles, liver, and brain. These findings suggest that EPPH is a potential therapeutic agent for treating exercise-induced fatigue.

**Abstract:**

Intense exercise is reported to induce physical and cognitive fatigue, but few studies have focused on treatments to alleviate fatigue. We hypothesized that the oral supplementation of enzymatic porcine placenta hydrolysate (EPPH) prepared using protease enzymes could alleviate exercise-induced fatigue in an animal model. The objectives of the study were to examine the hypothesis and the action mechanism of EPPH in relieving physical and cognitive fatigue. Fifty male Sprague–Dawley rats aged 8 weeks (body weight: 201 g) were classified into five groups, and rats in each group were given oral distilled water, EPPH (5 mg nitrogen/mL) at doses of 0.08, 0.16, or 0.31 mL/kg body weight (BW)/day, or glutathione (100 mg/kg BW/day) by a feeding needle for 5 weeks, which were named as the control, L-EPPH, M-EPPH, H-EPPH, or positive-control groups, respectively. Ten additional rats had no intense exercise with water administration and were designated as the no-exercise group. After 2 weeks, the rats were subjected to intense exercise and forced swimming trial for 30 min once per week for an additional 4 weeks. At 5 min after the intense exercise, lactate concentrations and lactate dehydrogenase (LDH) activity in the serum and the gastrocnemius muscle were higher in the control group, whereas M-EPPH and H-EPPH treatments suppressed the increase better than in the positive-control (*p* < 0.05). Intense exercise decreased glycogen content in the liver and gastrocnemius muscle, and M-EPPH and H-EPPH inhibited the decrement (*p* < 0.05). Moreover, lipid peroxide contents in the gastrocnemius muscle and liver were higher in the control group than in the M-EPPH, H-EPPH, positive-control, and no-exercise groups (*p* < 0.05). However, antioxidant enzyme activities such as superoxide dismutase (SOD) and glutathione peroxidase (GSH-Px) were opposite to the lipid peroxide contents. Hypothalamic corticosterone and hippocampal mRNA expressions of tumor necrosis factor (TNF)-α and IL-1β were higher. However, hippocampal brain-derived neurotrophic factor (BDNF) mRNA expression and protein contents were lower in the control group than in the positive-control group. M-EPPH, H-EPPH, and positive-control suppressed the changes via activating hippocampal cAMP response element-binding protein phosphorylation, and H-EPPH showed better activity than in the positive-control (*p* < 0.05). In conclusion, EPPH (0.16–0.31 mL/kg BW) intake reduced exercise-induced physical and cognitive fatigue in rats and could potentially be developed as a therapeutic agent for relieving fatigue in humans.

## 1. Introduction

Fatigue, often associated with low energy, is an overall feeling of physical and/or mental tiredness. Physical fatigue results from muscle fatigue due to excessive physical activity, whereas mental stress, anxiety, and insufficient sleep induce mental fatigue, in turn affecting cognitive ability [1]. Mental fatigue causes a deterioration in physical fatigue, thereby exacerbating the condition of fatigue. People seek medical assistance to alleviate the condition; however, its causes remain unclear and complex [2]. While excessive physical activity is the most known cause of fatigue, it has also been associated with several disease conditions. These include autoimmune diseases, anxiety disorders, anemia, cancer, chronic fatigue syndrome, fibromyalgia, depression, metabolic disorders, infectious disease, and other conditions involving chronic low-level inflammation [2,3]. In one study, among those diagnosed with fatigue, 19.4%, 16.5%, and 8.2% were diagnosed with musculoskeletal, psychological, and definitive physical conditions, respectively, and the remaining 55.9% could not be diagnosed. However, the underlying cause of the fatigue could not be ascertained [4]. Based on this study, physical fatigue could be considered one of the main types.

Although moderate exercise is known to improve insulin sensitivity and glucose utilization in the muscles, prolonged and strenuous exercise induces fatigue, resulting in reduced exercise capacity [5,6]. Exercise-induced fatigue is defined as an acute impairment of exercise performance and is intertwined with physical and mental states [1]. However, its etiology has been linked to multiple factors, such as lactate accumulation, insufficient glucose supply, increased oxidative stress, skeletal muscle mitophagy, and inflammation, although it is not entirely understood [7,8]. Exercise-induced mental fatigue is initially induced due to increased catecholamine in the brain, which decreases cognitive ability and exercise capability [9]. Furthermore, the dopaminergic and serotonergic systems play a critical role in cognitive fatigue [1], and the brain-derived neurotrophic factor (BDNF) expression is involved in reducing fatigue, possibly through dopaminergic receptor activation [10]. Exercise-induced cognitive fatigue does not result from a single central mechanism but from impaired integrations in neurotransmitter alterations in the multiple brain regions [11]. Therefore, exercise-induced fatigue needs to be mitigated at both the physical and cognitive levels to reduce its adverse effects.

Various foods to alleviate fatigue have been commercialized in the market. They aim to reduce oxidative stress, inflammation, and catecholamine levels and improve dopaminergic receptor activation and neurotropic factor expression [12,13]. Most contain proteins, peptides, amino acids, taurine, oligosaccharides, polyphenols, vitamin C, and caffeine [14,15,16]. These products are not very efficacious, and some have adverse effects [14]. The placenta contains many nutrients to enhance fetal growth during pregnancy, including human functional components. After delivering a baby, the placenta has been used as a traditional medicine in Asian countries [17]. It is known to alleviate various diseases, including dyslipidemia, osteoporosis, liver dysfunction, and gynecological disorders. Porcine placenta extracts have been reported to have health benefits by improving immunity and cell regeneration and reducing oxidative stress and inflammation [18,19].

The primary components of porcine placenta extracts are amino acids and peptides [18]. The amino acids and small peptides retained in the porcine placenta hydrolysates serve as active compounds for therapeutic use [18]. The present study hypothesized that oral supplementation of enzymatic porcine placenta hydrolysates (EPPH) prepared by protease enzymes could alleviate exercise-induced physical and cognitive fatigue in an animal model. The objective of the present study was to test the hypothesis and clarify the action mechanism of EPPH. The action mechanism of EPPH was assessed for how it reduced skeletal muscle, liver, and brain damage by promoting lactate removal and decreasing oxidative stress and inflammation. A previous study also evaluated the rat model of exercise-induced fatigue induced by running on a treadmill with a load for 30 min [20].

## 2. Materials and Methods

### 2.1. EPPH Preparation

The EPPH was prepared by Ubio (Gangneung, Republic of Korea) as follows. The porcine placenta was washed with saline (0.9% NaCl solution) three times to remove its blood and debris. The porcine placenta was then crushed and hydrolyzed with a proteolytic enzyme. After inactivating the protease by heating, it was filtered after ethanol precipitation and evaporated. It was then followed by adsorption with activated carbon. After additional filtration, the EPPH was autoclaved. The EPPH was mainly composed of amino acids and peptides of mass 2–5 kD. The amino acid contents in the EPPH were assessed using an amino acid analyzer (S433; SYKAM GmbH, Eresing, Germany). Peptide contents were calculated using the equation: (total nitrogen amount—total amino acids amounts)/total nitrogen amounts.

### 2.2. Animal Care and Experimental Design

Sixty male Sprague–Dawley (SD) rats aged seven weeks (201 ± 4.89 g) were purchased from Daehan Bio Inc. (Eum-Sung, Republic of Korea) and were lodged in individual stainless-steel cages in an animal facility for seven days. They were fed rodent feed (Samyang Feed Co., Gangwon, Republic of Korea) and water ad libitum, and the facility was kept under controlled conditions of 23 °C and 12 h light/dark cycle. All animal care procedures conformed with the Guide for the Care and Use of Laboratory Animals (8th edition) published by National Academy Press, Washington, DC, in 2011. The animal study was approved by the Institutional Animal Care and Use Committee of Hoseo University (HSIACUC-201932). 

**Figure 1:**
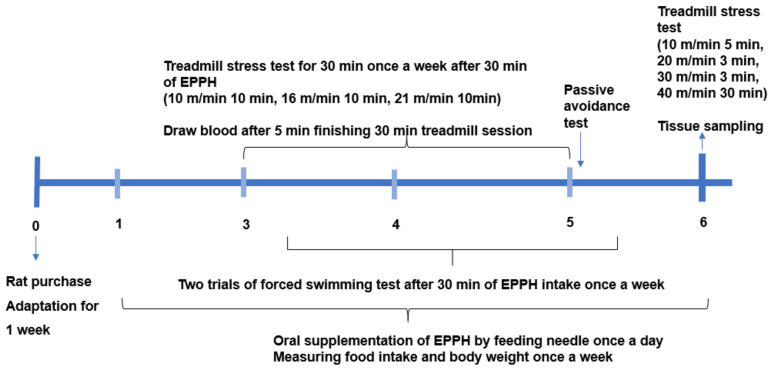
Experimental design. Rats had oral supplementation of EPPH (0.08, 0.16, and 0.31 mL/kg body weight) by a feeding needle once per day for two weeks, and from the third week, rats were given intense exercise using a treadmill protocol for 30 min after 30 min of being given EPPH. However, rats in the no-exercise group did not have intense exercise. The treadmill exercise was conducted for two weeks according to the protocol 10 m/min for 10 min, 16 m/min for 10 min, and 21 m/min for 10 min. Two days after the treadmill exercise, they were given a forced swimming test 30 min after EPPH supplementation. In the fourth week after starting the treadmill, the final treadmill test was performed according to the protocol of 10 m/min for 5 min, 20 m/min for 3 min, 30 m/min for 3 min, 40 m/min for 30 min, and after 5 min, rats were sacrificed for collecting their tissues.

The experimental design is presented in Figure 1. Sixty rats were divided into six groups, with 10 rats per group: control, L-EPPH, M-EPPH, H-EPPH, positive-control, and no-exercise. The control and no-exercise groups were administered distilled water orally. The EPPH (5 mg total nitrogen contents/mL) was orally and daily administered at doses of 0.08, 0.16, and 0.31 mL (2.5, 5.0, and 9.7 mg amino acid equivalent) per kg body weight for the three EPPH groups. Exercise-induced fatigue is involved in increased oxidative stress, and glutathione acts as endogenous antioxidant defense and detoxification. Glutathione intake improved muscle fatigue induced by prolonged exercise [21]. Glutathione (100 mg/kg body weight/day) was administered to the positive-control group with a feeding needle for six weeks. EPPH and glutathione were dissolved in 1 mL of distilled water for administration, and rats in the control group had 1 mL of distilled water without any agent by a feeding needle. From the third week of the assigned treatment, the 12 h fasting rats were made to run on a treadmill to achieve exercise-induced fatigue or forced swimming every week at 30 min after being administered water, the EPPH, or glutathione treatment. However, the rats in the no-exercise group did not have intense exercise. Blood was collected from the tail vein 5 min after finishing the 30 min treadmill/swimming sessions.

### 2.3. Diet Preparation for Rats

During the experimental period, all rats freely consumed a high-fat diet based on a modified polyphenol-free AIN-93 semi-purified method. High-fat diets have been reported to increase fatigue and daytime sleepiness [22]. The high-fat semi-purified diet contained 37 energy percent (En%), carbohydrates (cornstarch and sucrose), 20 En% protein (casein), 4 En% corn oil, 39 En% lard (CJ Co., Seoul), with added vitamins and minerals. The rodent diet was then re-sieved to mix the ingredients well and stored at 4 °C. Each diet had equivalent nutrient composition and energy (5.1 kcal/g). All the rats also had free access to water.

### 2.4. Exercise-Induced Fatigue

Rats in the exercise groups were pre-trained on the treadmill (Scitech Korea Inc., Seoul, Republic of Korea) for 15 min daily at 10 m/min speed for three successive days to adapt to the apparatus prior to initiating the EPPH administration. After the third week of the EPPH administration, the 12 h fasted rats were orally administered with water, EPPH, or glutathione, and 30 min later, they were subjected to 30 min running session on the treadmill once a week. The 30 min running session was composed of 10 m/min for 10 min, 16 m/min for 10 min, and 21 m/min for 10 min, successively. Five minutes after finishing the treadmill test, blood was collected from the tail tip of the rats to measure fatigue biomarkers. On the final day of the intervention, the 12 h fasted rats had an oral administration of water, EPPH, or glutathione, and then 30 min later, they ran a 41 min treadmill session at 10 m/min for 5 min, 20 m/min for 3 min, 30 m/min for 3 min, and 40 m/min for 30 min. When the rats refused to run, they were made to run by gently nudging them with a stick.

### 2.5. Forced Swimming Test

Two days later, after the treadmill exercises in the 3rd, 4th, and 5th weeks, the rats had no food for 8 h and performed a 10 min pretest in a clear acrylic cylinder, 60 cm in height and 30 cm in width, filled with 45 cm water at 24 ± 1 °C. Twenty-four hours after the pretest, the rats were given a 5 min forced swimming test in the cylinder, and the movement was scored for mobile (like swimming and climbing) or immobile behaviors. The movement was videotaped during the trials, and the researcher randomly and blindly selected the movement type. The researcher evaluated the videos of the 5 min session and scored the total time of active and passive behaviors. The results were designated as the ‘active’ time during the 5-min session. When the animal had longer active time during the forced swimming session, it was considered less depressed.

### 2.6. Passive Avoidance Test

A passive avoidance apparatus composed of dark/light shuttle boxes was used to evaluate memory and learning. In the last week of the interventions, rats were allowed to spontaneously enter the dark box when placed in the light shuttle box of the apparatus [23]. The latency time to enter the dark room was measured to determine the short-term memory in each trial. A weak electrostimulation (75 V, 0.2 mA, 50 Hz) was delivered to the feet of the rat when it entered the dark box to teach it not to enter the dark room in two training sessions with an eight-hour term. At 16 h after the second trial, the third trial was repeated in the same manner as the previous trials but without electrostimulation. Latency was measured up to a maximum of 600 s. The longer the latency time, the better the memory function.

### 2.7. Tissue Collection and Assays

When finishing the intense exercise in the 6th week, the rats were anesthetized with ketamine and xylazine. After anesthetizing the animals with a mixture of ketamine and xylazine (100 and 10 mg/kg body weight, respectively), blood was drawn from the inferior vena cava, and tissues were immediately collected. The liver, spleen, quadriceps and gastrocnemius muscle, epididymal and retroperitoneal fat, and brain were dissected, weighed, and frozen at −70 °C. The liver and spleen indices were calculated as their percentages of body weight (% wt).

The hippocampi were divided into two sections: one portion was lysed with a radioimmunoprecipitation assay (RIPA) buffer containing protease inhibitors, and the other was randomly selected and lysed with Trizol reagent (Invitrogen, Rockville, MD, USA) for extracting the total RNA. The hippocampal RIPA buffer lysates were centrifuged at 5000× *g* at 4 °C for 10 min. The supernatants were used to measure the lipid peroxide and cholesterol contents by a colorimetry method using a lipid peroxidation (malondialdehyde (MDA)) assay kit (Abcam, Cambridge, UK) and cholesterol kit (Asan Pharmaceutics). The supernatants were deproteinized with 1.5 N perchloric acid, and the glycogen content was calculated from the glucose concentrations from the glycogen hydrolyzed by α-amyloglucosidase in an acid buffer [24]. The glucose concentrations were measured using a glucose kit (Asan Pharmaceutics, Seoul, Republic of Korea). Triglycerides were extracted from the hippocampus with a chloroform–methanol (2:1, *v:v*) solution and resuspended in pure chloroform [25]. Lactate concentrations and lactate dehydrogenase (LDH) activity in the serum and skeletal muscle lysates were determined with their corresponding kits (DoGenBio, Seoul, Republic of Korea). Serum total antioxidant capacity, superoxide dismutase (SOD), and glutathione peroxidase (GSH-Px) activity were measured with the respective kits (DoGenBio, Seoul, Republic of Korea). The triglyceride content in the chloroform was measured using a triglyceride colorimetric kit (Asan Pharmaceutics, Seoul, Republic of Korea). Serum tumor necrosis factor-alpha (TNF-α) and interleukin (IL)-1β concentrations were measured using enzyme-linked immunosorbent assay (ELISA) kits (eBioscience; San Diego, CA, USA). Serum aspartate transaminase (AST), alanine transaminase (ALT), and creatine kinase activities, and serum blood urinary nitrogen (BUN) concentrations were also assayed with the respective kits (Asan Pharmaceutics, Seoul, Republic of Korea). 

### 2.8. Quantitative Real-Time PCR

Complementary DNA (cDNA) was produced using a mixture of isolated total RNA from the brain and liver with superscript III reverse transcriptase and high-fidelity Taq DNA polymerase (1:1:1, *v:v:v*) using a polymerase chain reaction (PCR) machine. The cDNA was mixed with the primers for the genes of interest and an SYBR Green mix to determine the expressions of the designated genes using a real-time PCR machine (BioRad Laboratories, Hercules, CA, USA) [25]. The primers for the BDNF, TNF-α, IL-1β, and β-actin were used (Supplementary Table S1). The gene expression levels were quantitated using the comparative cycle of threshold (CT) method [24].

### 2.9. Statistical Analyses

The sample size for testing the effects of EPPH on exercise-induced fatigue was determined using the G power program (power = 0.85 and effect size = 0.50). Statistical analysis was performed using the SAS version 7 (SAS Institute; Cary, NC, USA) program, and the results were expressed as the mean ± standard deviation (SD). Univariate analysis was used to check the normal distribution of variables, and one-way analysis of variance (ANOVA) was conducted to determine the significance of the groups in each variable when it was normally distributed. In one-way ANOVA, multiple comparisons were performed with Tukey’s test when the variable was significantly different among the groups. Statistical significance was accepted at *p* < 0.05.

## 3. Results

### 3.1. Amino Acid Composition of EPPH Product

EPPH product contained all 20 amino acids, of which 16 were measured. It included high proportions of glycine, arginine, alanine, leucine, and lysine; oligo-peptides contained 57.2 ± 15.3 % (Table 1).

### 3.2. Body Weight, Organ Index, and Serum Glucose Concentrations

The final body weight and weight gain during the 6 weeks were not significantly different between the control, positive-control, and no-exercise groups. However, they were lower in the M-EPPH and H-EPPH groups than in the control group (Table 2). Food intake was not significantly different among the groups, and food efficiency calculated by dividing body weight gain by food intake was significantly lower in the M-EPPH group compared to the others (Table 2). Protein intake was about 5 g/day in each group, and there was no significant difference among the groups (Table 2). The EPPH intake was much less than protein intake, and its effect might not be linked to the changes in the amino acid pool. Epididymal and retroperitoneal fat and visceral fat mass were higher in the positive-control group than in the control and positive-control group, while it was lower in the M-EPPH and H-EPPH groups (Table 2). However, muscle mass was not significantly different among the groups. The liver and spleen indices, calculated as the percentage of the organ weight based on the body weight, were higher in the control group than in the positive-control group, while all dosages of EPPH inhibited the increase in their indices, and M-EPPH decreased the spleen index compared to the other groups (Table 2). M-EPPH and H-EPPH improved liver and spleen indices impaired by exercise-induced fatigue to levels similar to the positive-control group.

**Figure 2:**
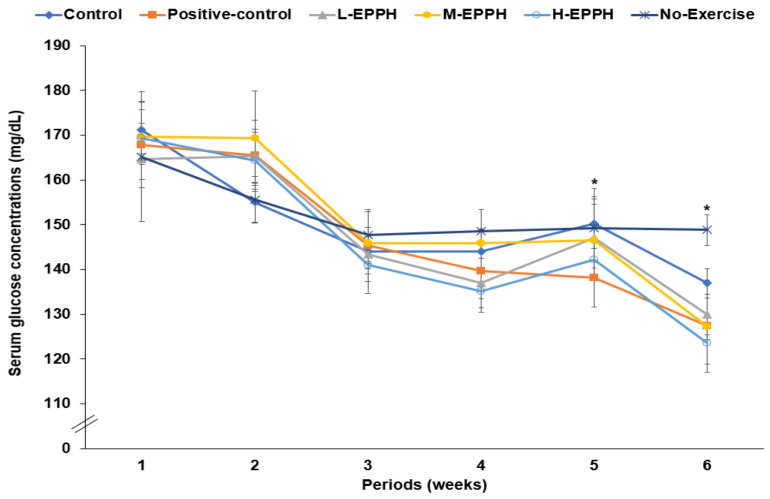
Fasting serum glucose concentrations before exercise-induced fatigue during the experimental periods. Dots and error bars in each period represent means ± standard deviation (SD; *n* = 10). The rats with exercise-induced fatigue had oral administration of distilled water for the control group, porcine placenta enzyme hydrolysates (EPPH) dissolved in distilled water for experimental groups, and glutathione (100 mg/kg body weight) for the positive-control group. EPPH (0.08, 0.16, and 0.32 mL/kg body weight) was orally provided with a feeding needle for the L-EPPH, M-EPPH, and H-EPPH groups, respectively, for 6 weeks. The rats were designated as the no-exercise group without exercise-induced fatigue and oral administration of distilled water. * Significantly different among groups in one-way ANOVA at *p* < 0.05.

The serum glucose concentrations in the fasting state were not significantly different among the groups in the first and second weeks. However, after achieving exercise-induced fatigue, the serum glucose concentrations in the fasting state were lower in the positive-control, M-EPPH, and H-EPPH groups than in the control group (Figure 2, Table 3). It was related to the beneficial impact of exercise on serum glucose concentrations, even with intense exercise-induced fatigue. The fasting serum insulin concentrations increased in the control and no-exercise groups compared to the positive-control and EPPH groups. The threshold homeostasis model assessment of insulin resistance (HOMA-IR) levels (calculated with the serum glucose and insulin concentrations) was much higher in the control group. However, it decreased in the positive-control, L-EPPH, and M-EPPH groups compared to the control group (Table 3). The results were related to lowering insulin resistance and oxidative stress by glutathione and EPPH.

### 3.3. Exercise-Induced Fatigue in the Skeletal Muscles

**Figure 3:**
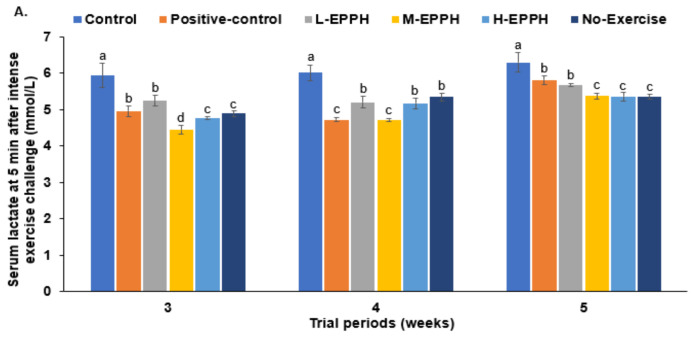

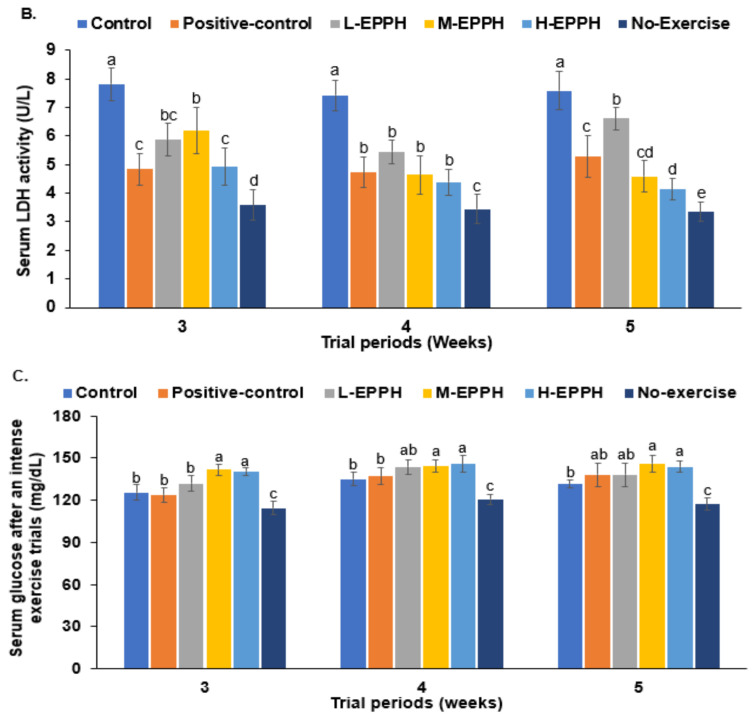
Metabolites related to exercise-induced fatigue at 5 min after intense exercise; (**A**) serum lactate concentrations; (**B**) LDH activity in the serum; (**C**) serum glucose concentrations. Bars and error bars represent means ± standard deviation (SD; *n* = 10). The rats with exercise-induced fatigue had oral administration of distilled water for the control group, porcine placenta enzyme hydrolysates (EPPH) dissolved in distilled water for experimental groups, and glutathione (100 mg/kg body weight) for the positive-control group. EPPH (0.08, 0.16, and 0.32 mL/kg body weight) was orally provided with a feeding needle for the L-EPPH, M-EPPH, and H-EPPH groups, respectively, for 6 weeks. The rats that had no exercise-induced fatigue and oral administration of distilled water were designated the no-exercise group. ^a,b,c,d^ Different letters on the bars indicate significant differences among the groups by Tukey test at *p* < 0.05.

At 5 min after the intense-exercise challenge, serum lactate concentrations were much higher in the control group than in the no-exercise group. They were lower in the positive-control and EPPH groups in the 3rd, 4th, and 5th weeks (Figure 3A). The serum lactate concentrations were the lowest in the M-EPPH groups after all three exercise-induced fatigue trials. The results indicated that lactate production was lower or elimination was much higher with M-EPPH and positive-control groups than with the control group.

The serum LDH activity was much higher in the control group than in the positive-control group. While serum LDH activity decreased in the positive-control and EPPH groups, it was still higher than in the positive-control group in the 3rd, 4th, and 5th weeks (Figure 3B). In the 5th week, it was lower in the H-EPPH group than in the positive-control. This indicated that EPPH at all doses and glutathione treatments lowered lactate production more than the control after the intense-exercise challenge.

However, serum glucose concentrations increased in the control group compared to the positive-control group, while they were elevated in the M-EPPH and H-EPPH groups in the 3rd, 4th, and 5th weeks (Figure 3C). This indicated that lactate was rapidly converted to glucose in the M-EPPH and H-EPPH groups.

At the last intense exercise session in the sixth week, serum lactate concentrations were higher in the control group than in the positive-control group. They decreased in descending order of the control, L-EPPH, M-EPPH, positive-control, H-EPPH, and no-exercise (Table 3). Serum cortisol concentrations were shown to be much higher in the control group than in the no-exercise group but were lower in the positive-control, M-EPPH, and H-EPPH groups (Table 3).

The total serum antioxidant capacity decreased in the control group compared to the no-exercise group. It did not decrease in the M-EPPH, H-EPPH, and positive-control groups compared to the control group (Table 3). Its decrement was associated with increased SOD and GSH-Px activities in rats given EPPH and glutathione after intense exercise. The activities in the no-exercise group were not as high as those in the EPPH and positive-control groups. However, the total antioxidant capacity was high in the no-exercise group (Table 3). These results indicate that intense exercise elevated oxidative stress, which was rapidly eliminated by activating antioxidant enzymes in the EPPH and positive-control groups. In addition to oxidative stress, serum TNF-α and IL-1β concentrations and inflammatory indices were higher in the control group than in the no-exercise group, while they were suppressed in the M-EPPH, L-EPPH, and positive-control groups (Table 3). Intense exercise increases oxidative stress, which could induce muscle and liver damage. Serum creatinine and BUN concentrations were elevated in the control group compared to the no-exercise group, whereas M-EPPH, H-EPPH, and positive-control inhibited their increase (Table 3).

Serum creatine kinase activity also increased in the control group compared to no-exercise, and M-EPPH, H-EPPH, and positive-control suppressed the increase. These changes indicated that intense exercise increased muscle damage, and EPPH and glutathione could inhibit it (Table 3). Intense exercise also elevated serum AST and ALT activities and the liver damage indices, but EPPH and glutathione intake decreased the indices after intense exercise (Table 3).

In addition to serum concentrations, lactate concentration in the gastrocnemius was higher in the control group than in the no-exercise group. EPPH reduced the lactate increment in a dose-dependent manner after intense exercise in the 6th week, and its increase was lower in M-EPPH and H-EPPH than in the positive-control (Table 4). LDH and creatine kinase activities were also higher in the control group than in the positive-control group, but they decreased in M-EPPH and H-EPPH compared to the control (Table 4). Gastrocnemius lipid peroxide content, one of the physical fatigue-related factors, dose-dependently decreased with EPPH compared to the control, and it was lower in H-EPPH than in the positive-control. The MDA reduction by EPPH was linked to increased SOD and GSH-Px in rats with exercise-induced fatigue (Table 4). SOD and GSH-Px activities were lower in the control group than in the positive-control, and EPPH groups increased their activities the most in H-EPPH (Table 4). They did not increase in the no-exercise group as much as H-EPPH since the rats in the no-exercise group did not have induced oxidative stress due to no intense exercise. In addition to oxidative stress, glycogen contents in the gastrocnemius were lower in the control group than in the no-exercise group, and it increased in the order of the control, L-EPPH, no-exercise, M-EPPH, positive-control, and H-EPPH groups (Table 4).

The glycogen and MDA contents in the liver were similar to those in the gastrocnemius muscle (Table 4). The SOD and GSH-Px activities were higher in the control group than in the no-exercise group, and they increased in the order of the control, L-EPPH, no-exercise, M-EPPH, positive-control, and H-EPPH (Table 4). The intense exercise increased MDA contents and decreased glycogen in the control group compared to the no-exercise group, indicating that the intense exercise elevated oxidative stress and decreased glycogen deposition in the gastrocnemius (Table 4). Therefore, the SOD and GSH-Px activities were not as high in the no-exercise group compared to the positive-control and H-EPPH treatment groups, although MDA contents in the no-exercise were lower than those in the positive-control and H-EPPH groups.

### 3.4. Forced Swimming and Passive Avoidance Tests

**Figure 4:**
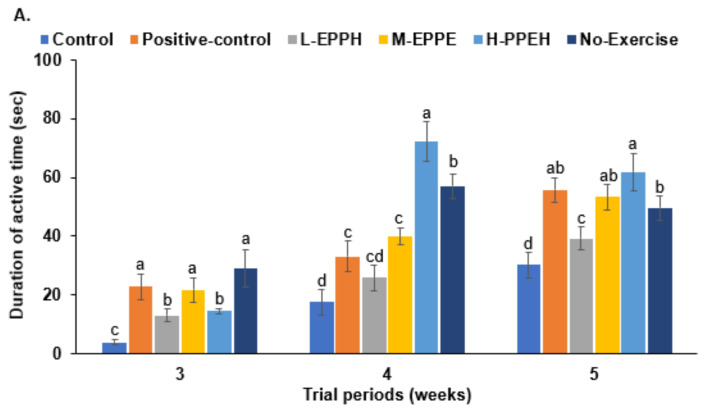

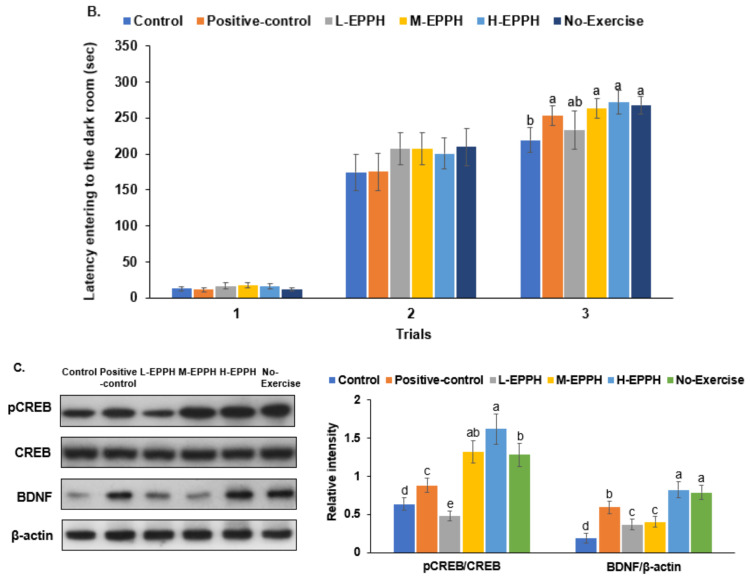
Brain fatigue; (**A**) the active time during forced swimming; (**B**) latency entering the dark room; (**C**) hippocampal Western blots. Bars and error bars represent means ± standard deviation (SD; *n* = 10). The rats with exercise-induced fatigue had oral administration of distilled water for the control group, porcine placenta enzyme hydrolysates (EPPH) dissolved in distilled water for experimental groups, and glutathione (100 mg/kg body weight) for the positive-control group. EPPH (0.08, 0.16, and 0.32 mL/kg body weight) was orally provided with a feeding needle for the L-EPPH, M-EPPH, and H-EPPH groups, respectively, for 6 weeks. The rats that had no exercise-induced fatigue and oral administration of distilled water were designated the no-exercise group. ^a,b,c,d^ Different letters on the bars indicate significant differences among the groups by Tukey test at *p* < 0.05.

In the 3rd, 4th, and 5th weeks, the active time duration during the forced swimming was much higher in the positive-control and no-exercise groups than in the control group, while EPPH administration also increased the active time (Figure 4). In the 4th and 5th weeks, the active periods were higher in the H-EPPH group than in the no-exercise group. Long-term EPPH thus inhibited exercise-induced fatigue.

At 1 h after intense exercise, the third passive avoidance test was conducted. When a rat develops fatigue, its short memory is impaired. In the first and second trials, the latency time entering the dark room did not significantly differ among the groups (Figure 4B). In the third trial, it was shorter in the control group than in the no-exercise group, while it increased in the positive-control, M-EPPH, and H-EPPH groups, compared to the control group (Figure 4B). This indicated that EPPH and glutathione could suppress the short-term memory impairment induced by intense exercise.

### 3.5. Exercise-Induced Cognitive Fatigue in the Brain

Lipid peroxide content in the hypothalamus was higher in the control group than in the no-exercise group, and its increase was suppressed in the positive-control and EPPH groups (Table 5). The contents in the M-EPPH and H-EPPH groups were similar to the no-exercise group. An increase was also seen in the levels of the inflammatory indices, TNF-α and IL-1β. The TNF-α and IL-1β contents were higher in the control group than in the no-exercise and decreased in the M-EPPH and H-EPPH groups, similar to the no-exercise group (Table 5). The decrease in the hippocampal inflammatory indices was higher in the positive-control than in the M-EPPH and H-EPPH.

Increased lipid peroxides and inflammatory indices were linked to elevated hypothalamic corticosterone mRNA expression. Hypothalamic corticosterone is associated with activating the hypothalamus–pituitary–adrenal (HPA) axis. Corticosterone mRNA expression was much higher in the control group than in the no-exercise and decreased in the positive-control and EPPH groups (Table 5). Furthermore, hippocampal BDNF mRNA expression was lower in the control group than in the no-exercise group, and M-EPPH, H-EPPH, and positive-control suppressed its decrease (Table 5). In the hippocampus, TNF-α and IL-1β expression was higher in the control group than in the no-exercise, and M-EPPH and H-EPPH decreased their expression (Table 5).

Intense exercise lowered cAMP response element-binding protein (CREB) phosphorylation in the hippocampus and elevated it in the positive-control and EPPH groups (Figure 4C). The highest elevation was seen in the M-EPPH and positive-control groups. BDNF protein content was lower in the control group than in the no-exercise group, whereas EPPH and positive-control suppressed its decrease (Figure 4C). Similar to the BDNF mRNA expression, hippocampal BDNF protein content did not increase in the positive-control as much as in the M-EPPH and H-EPPH groups (Figure 4C). These results suggested that intense exercise elevated the HPA axis by elevating corticosterone expression, which was related to increased oxidative stress and inflammation. 

## 4. Discussion

It is well known that physical exercise improves overall health by enhancing muscle growth and strength, improving the cardiovascular system, and losing body fat [26,27]. However, intense and excessive exercise could exceed the body’s ability to recover, thus harming health [9]. The mechanism of exercise-induced fatigue has yet to be entirely understood. However, it generally involves metabolite accumulation, insufficient energy supply, dysregulation of neurotransmitters and the HPA axis, oxidative stress, and inflammation. Accumulation of exercise-induced fatigue results not only in decreased work efficiency but also in immune and endocrine dysregulation [9]. The present study investigated the effects of EPPH on exercise-induced fatigue and its mechanism in rats. The results suggest that EPPH reduces lactate accumulation in the skeletal muscles by potentiating its conversion into glucose after intense exercise and increases glycogen deposition in the skeletal muscles and liver. Furthermore, it reduces cognitive fatigue by potentiating CREB phosphorylation to elevate BDNF expression in the hippocampus. In addition, EPPH decreases oxidative stress and inflammation involved in exercise-induced physical and cognitive fatigue. Therefore, EPPH can be a potential intervention for alleviating exercise-induced physical and mental fatigue, thus contributing to improving the beneficial effects of exercise.

Functional foods can assist in the management of exercise-induced physical and cognitive fatigue by providing energy or boosting compounds such as adenosine triphosphate (ATP), glucose, creatine, glutamate, L-carnitine, and betaine, antioxidants such as vitamin C and E, coenzyme Q, phytochemicals, and neurotransmitter modulators such as branched-chain amino acids and caffeine [28]. In our study, EPPH was prepared by removing blood from the porcine placenta and hydrolyzing it with protease, and the final hydrolysate comprised amino acids and small peptides. Its primary amino acids were glycine, glutamate, arginine, alanine, leucine, and lysine. EPPH prepared in this manner potentially modulated exercise-induced fatigue by providing energy and antioxidant-rich constituents and modulating the neurotransmitter function. Several earlier studies have demonstrated the benefits of placenta hydrolysates in alleviating liver damage and improving menopausal symptoms in animal models [18,29]. The leucine–glycine dipeptide from the porcine placenta has been shown to improve exercise-induced physical fatigue by reducing proinflammatory cytokines and oxidative stress in animal models [19,29,30]. Previous studies have shown that the oral administration of taurine, glutamate, or other amino acids decreases blood lactate accumulation and neuromuscular fatigue in healthy participants [31,32]. Therefore, EPPH intake before intensive exercise may decrease exercise-induced physical fatigue.

Exercise-induced fatigue increases LDH and creatine kinase activities in the muscles and blood to produce lactate and creatinine, contributing to a decrease in the muscle and blood pH. In addition, increased BUN indicates elevated muscle catabolism and damage [33]. Lactate is converted into glucose in the liver, which is then circulated into the muscles through blood [34]. Elevated lactate clearance appears to be the primary factor in alleviating exercise-induced fatigue. The acute increase in serum glucose concentrations suggests that an increased Cori cycle activity removes lactate and supplies glucose to the skeletal muscles [34]. In the present study, EPPH elevated serum glucose concentrations during intense exercise. EPPH prevented the accumulation of biochemical markers responsible for exercise-induced fatigue in a dose-dependent manner. Of the various doses studied, M-EPPH was found to provide significant benefits in improving lactate production and clearance, thus contributing to the prevention of muscle and liver damage in the present study.

In addition to decreased lactate, BUN, and creatinine concentrations in the blood and skeletal muscles, the glycogen content in the skeletal muscles and liver indicated efficient energy utilization to alleviate exercise-induced fatigue, thus increasing sustainable active time during intense exercise [35]. The high glycogen content in the skeletal muscles and liver suggests efficient lactate conversion and energy availability, enabling a person to tolerate intense exercise. In the present study, EPPH increased glycogen synthesis to reduce exercise-induced fatigue and increase the active periods during forced swimming.

Chronic physical and cognitive fatigue is also related to elevated oxidative stress and inflammation, damaging blood and tissues, especially skeletal muscles and the liver [36]. Exhaustive exercise damages the tissues by elevating the reactive oxygen species (ROS) beyond the antioxidant capacity. The body has antioxidant systems such as SOD, GSH-Px, and catalase to remove ROS. In moderate exercise, the antioxidant system eliminates ROS efficiently [26]. Antioxidant supplementation has also been shown to reduce oxidative stress and fatigue symptoms [37]. EPPH has both antioxidant and antibacterial properties. However, the properties vary according to the hydrolysis process and used proteases [38]. The EPPH used in the present study reduced lipid peroxides in the blood and skeletal muscles by activating the antioxidant system and increasing SOD and GSH-Px activity. Along with oxidative stress, proinflammatory cytokines were observed to be elevated due to exercise-induced fatigue in the present study. Inflammation is known to be correlated with fatigue symptoms [39]. In the present study, exercise-induced fatigue increased proinflammatory cytokines, TNF-α and IL-1β, and EPPH reduced them. IL-1β induces IL-6 production, linked to intense exercise [40], and exercise-induced fatigue may increase IL-6 contents. Low-grade inflammation resulting from reduced cellular energy availability, and an imbalance in energy expenditure induces fatigue [41,42]. Anti-inflammatory diets and supplements, such as whole grains, vegetables, selenium, and omega-3 fatty acids, can alleviate physical and cognitive fatigue [43,44]. Similarly, EPPH administration prevented an increase in inflammation and oxidative stress due to exhaustive exercise, thus contributing to reducing exercise-induced fatigue.

Intense exercise generates both physical and cognitive fatigue [45], although long-term moderate exercise improves cognitive functions [46]. However, the mechanism of intense exercise to induce cognitive fatigue has remained controversial [45,47,48]. The present study demonstrated that EPPH improved physical and cognitive fatigue induced by exhaustive exercise. Cognitive fatigue was related to decreased glucose concentrations and neurotransmitter alteration, increased proinflammatory cytokines, and hypoxemia in the brain [9,47]. It is associated with cognitive impairment and low alertness [49]. The CREB protein in the forebrain is involved in neuronal plasticity, memory, and social behavior, and its phosphorylation activates BDNF expression to increase neurogenesis and improve memory function [49]. The ingestion of some herbs, such as *Akebia Quinata* Decaisne extract, has been shown to attenuate cognitive fatigue induced by chronic restraint stress by potentiating CREB phosphorylation and increasing BDNF and tropomyosin receptor kinase B (TrkB) expression in mice [50]. The present study also demonstrated that intense exercise attenuated CREB phosphorylation and BDNF expression to reduce memory function and exacerbated some depressive conditions in the forced swimming test. EPPH suppressed exercise-induced cognitive fatigue by increasing CREB phosphorylation and BDNF expression and decreasing the hippocampal TNF-α and IL-1β content. EPPH action may be related to increased glucose supply and reduced oxidative stress and inflammation in the brain.

The present study had the strength to show that EPPH intake could reduce exercise-induced physical and cognitive fatigue in rats by promoting lactate utilization, reducing oxidative stress and inflammation in skeletal muscle and liver, and improving potent neuromodulatory activity. However, the EPPH efficacy needs to be confirmed in humans. Furthermore, its dosage of amino acids and peptides in EPPH was much lower than expected by simply increasing dietary protein. This suggests the presence of potent bioactive peptides that need to be isolated and evaluated. We need to be examined to be potential candidates for improving exercise-induced fatigue. This study has taken a novel approach in applying a traditional medicine prepared in a new way to mitigate exercised-induced mental and physical fatigue. This research expands our knowledge of how peptides from an uncommon source can be utilized to improve human performance. This study uses a novel approach that has received very little research attention.

## 5. Conclusions

Exercise-induced fatigue was linked to reduced lactate utilization and increased oxidative stress and inflammation in not only skeletal muscles but also in liver and brain tissues. EPPH rich in glycine, glutamate, arginine, alanine, leucine, lysine, and some oligo-peptides decreased the concentrations of fatigue-related metabolites, including lactate, BUN, and creatinine, as well as reduced liver damage and increased glycogen deposition in the liver and skeletal muscles after intense exercise. EPPH also inhibits the activation of the HPA axis (preventing an increase in serum cortisol) and elevates hippocampal BDNF mRNA expression and proteins by potentiating CREB phosphorylation. Furthermore, EPPH reduces oxidative stress and inflammation in the skeletal muscles, liver, and brain. Therefore, EPPH suppresses exercise-induced physical and cognitive fatigue by protecting against cell damage in the brain, liver, and skeletal muscles by suppressing oxidative stress and inflammation. These findings suggest that EPPH is a potential therapeutic agent for treating exercise-induced fatigue.

## Figures and Tables

**Table 1:** Amino acid contents in EPPH product.

	Content (mg/mL)		Content (mg/mL)		Content (mg/mL)
ASP	0.60 ± 0.25	ARG	1.43 ± 0.31	VAL	1.00 ± 0.40
SER	0.98 ± 0.20	TRE	0.59 ± 0.15	MET	0.62 ± 0.15
GLU	0.97 ± 0.19	ALA	1.45 ± 0.29	LYS	1.11 ± 0.23
GLY	1.30 ± 0.26	PRO	0.20 ± 0.10	ILU	0.84 ± 0.17
HIS	0.92 ± 0.18	TYR	0.36 ± 0.13	LUE	1.70 ± 0.34
PHE	0.61 ± 0.12				

Values are presented as means and standard deviations (*n* = 10).

**Table 2:** Body weight, food intake, and visceral fat mass after the 6-week intervention.

	Control (*n* = 10)	Positive-Control (*n* = 10)	L-EPPH (*n* = 10)	M-EPPH (*n* = 10)	H-EPPH (*n* = 10)	No-Exercise (*n* = 10)
Body weight (g)	429 ± 8.18 ^a^	425 ± 6.84 ^a^	429 ± 8.89 ^a^	416 ± 5.03 ^b^	418 ± 9.45 ^b^	420 ± 10.3 ^ab^
Weight gain during the 6-week intervention (g)	228 ± 11.5 ^ab^	220 ± 10.4 ^b^	233 ± 12.9 ^a^	208 ± 9.29 ^b^	213 ± 12.5 ^b^	219 ± 14.3 ^ab^
Food intake (g/day)	19.1 ± 0.40	18.4 ± 0.92	18.5 ± 0.68	19.5 ± 1.23	19.1 ± 1.56	18.9 ± 0.91
Protein intake (g/day)	5.17 ± 0.11	4.98 ± +0.25	5.01 ± 0.18	5.28 ± 0.33	5.17 ± 0.42	5.11 ± 0.25
Food efficiency (%)	12.2 ± 0.43 ^a^	12.3 ± 0.52 ^a^	12.7 ± 0.79 ^a^	11.2 ± 0.90 ^b^	11.8 ± 1.01 ^ab^	11.7 ± 0.83 ^ab^
Epididymal fat pads (g)	8.6 ± 0.48 ^b^	9.3 ± 0.86 ^a^	8.70 ± 0.86 ^ab^	7.05 ± 0.73 ^c^	7.94 ± 0.61 ^b^	8.01 ± 0.63 ^b^
Retroperitoneal fat (g)	10.1 ± 0.82 ^b^	11.5 ± 0.84 ^a^	9.58 ± 0.72 ^b^	8.12 ± 0.47 ^c^	8.71 ± 0.54 ^c^	8.48 ± 0.78 ^c^
Visceral fat mass (% of body weight)	4.36 ± 0.19 ^b^	4.89 ± 0.34 ^a^	4.26 ± 0.32 ^b^	3.65 ± 0.24 ^c^	3.97 ± 0.27 ^c^	3.93 ± 0.26 ^c^
Muscle mass (% of body weight)	2.43 ± 0.45	2.19 ± 0.29	2.35 ± 0.45	2.32 ± 0.33	2.39 ± 0.43	2.42 ± 0.44
Liver index (% of body weight)	3.91 ± 0.12 ^a^	3.68 ± 0.11 ^b^	3.75 ± 0.10 ^ab^	3.69 ± 0.07 ^b^	3.58 ± 0.10 ^b^	3.69 ± 0.09 ^b^
Spleen index (% of body weight)	4.33 ± 0.19 ^a^	4.41 ± 0.24 ^a^	4.24 ± 0.32 ^a^	3.63 ± 0.24 ^c^	3.91 ± 0.22 ^b^	3.91 ± 0.26 ^b^

Values represent means ± standard deviation (SD; *n* = 10). The rats with exercise-induced fatigue had oral administration of distilled water for the control group, porcine placenta enzyme hydrolysates (EPPH) dissolved in distilled water for experimental groups, and glutathione (100 mg/kg body weight) for the positive-control group. EPPH (0.08, 0.16, and 0.32 mL/kg body weight) was orally provided with a feeding needle for the L-EPPH, M-EPPH, and H-EPPH groups, respectively, for 6 weeks. The rats with no exercise-induced fatigue and oral administration of distilled water were designated as the no-exercise group. ^a,b,c^ Different superscript letters beside SD indicate significant differences among the groups by Tukey test at *p* < 0.05.

**Table 3:** Serum metabolic parameters related to exercise-induced fatigue at the 3rd and 5th week of the intervention.

	Control (*n* = 10)	Positive-Control (*n* = 10)	L-EPPH (*n* = 10)	M-EPPH (*n* = 10)	H-EPPH (*n* = 10)	No-Exercise (*n* = 10)
At fasting state in the 3rd week before the exercise challenge.						
Serum glucose (mg/dL)	137 ± 9.09 ^a^	127 ± 8.75 ^b^	130 ± 9.70 ^ab^	127 ± 9.64 ^b^	123 ± 8.41 ^b^	148 ± 6.38 ^a^
Serum insulin at fasting	1.96 ± 0.24 ^a^	1.50 ± 0.31 ^b^	1.75 ± 0.31 ^ab^	1.47 ± 0.27 ^b^	1.50 ± 0.19 ^b^	1.90 ± 0.20 ^a^
HOMA-IR	11.9 ± 1.11 ^a^	8.49 ± 0.87 ^c^	10.1 ± 1.27 ^b^	8.32 ± 0.91 ^c^	8.23 ± 0.76 ^c^	12.6 ± 1.30 ^a^
In the 5th week, after exercise-induced fatigue.						
Serum lactate at fasting state (mmol/L)	5.63 ± 0.25 ^a^	5.17 ± 0.12 ^b^	5.53 ± 0.16 ^a^	5.32 ± 0.10 ^b^	5.01 ± 0.09 ^c^	5.07 ± 0.14 ^c^
Serum cortisol (ng/mL)	129 ± 4.11 ^a^	120 ± 4.28 ^b^	126 ± 4.32 ^ab^	115 ± 4.53 ^bc^	113 ± 4.08 ^c^	104 ± 4.34 ^d^
Serum total antioxidant capacity	0.92 ± 0.05 ^c^	1.05 ± 0.05 ^a^	0.98 ± 0.05 ^b^	1.08 ± 0.04 ^a^	1.11 ± 0.06 ^a^	1.03 ± 0.06 ^b^
Serum SOD (U/mL)	1.35 ± 0.15 ^c^	1.72 ± 0.14 ^a^	1.44 ± 0.14 ^c^	1.63 ± 0.16 ^b^	1.76 ± 0.14 ^a^	1.48 ± 0.15 ^bc^
Serum GSH-Px (U/mL)	11.3 ± 0.85 ^d^	18.4 ± 1.78 ^a^	11.8 ± 0.91 ^d^	14.8 ± 1.07 ^c^	19.6 ± 1.19 ^a^	13.4 ± 1.05 ^c^
Serum TNF-α (pg/mL)	47.7 ± 1.13 ^a^	42.0 ± 1.28 ^c^	47.9 ± 1.44 ^a^	45.6 ± 1.06 ^b^	42.8 ± 0.83 ^c^	45.3 ± 1.35 ^b^
Serum IL-1β (pg/mL)	82.3 ± 1.05 ^a^	81.1 ± 1.23 ^a^	81.3 ± 1.17 ^a^	79.1 ± 0.60 ^b^	77.9 ± 0.70 ^c^	79.1 ± 0.94 ^b^
Serum BUN (mg/dL)	42.5 ± 1.67 ^a^	36.9 ± 1.60 ^c^	39.4 ± 1.37 ^b^	37.2 ± 0.89 ^bc^	36.5 ± 1.24 ^c^	36.2 ± 1.48 ^c^
Serum creatinine (mg/dL)	1.34 ± 0.06 ^a^	0.98 ± 0.10 ^c^	1.18 ± 0.13 ^b^	1.05 ± 0.15 ^bc^	0.98 ± 012 ^c^	1.02 ± 0.09 ^c^
Serum creatine kinase (U/L)	5.88 ± 0.57 ^a^	4.68 ± 0.24 ^b^	5.65 ± 0.41 ^a^	4.39 ± 0.41 ^b^	3.81 ± 0.36 ^c^	3.42 ± 0.40 ^c^
Serum AST (U/L)	72.5 ± 5.13 ^a^	62.9 ± 2.68 ^b^	61.5 ± 2.44 ^b^	52.0 ± 3.29 ^c^	46.3 ± 1.31 ^d^	52.8 ± 2.27 ^c^
Serum ALT (U/L)	63.3 ± 5.38 ^a^	53.4 ± 1.58 ^b^	51.2 ± 2.09 ^b^	39.0 ± 2.85 ^c^	33.3 ± 2.24 ^d^	35.8 ± 4.09 ^d^

Values are represented by means ± standard deviation (SD; *n* = 10). The rats with exercise-induced fatigue had oral administration of distilled water for the control group, porcine placenta enzyme hydrolysates (EPPH) dissolved in distilled water for experimental groups, and glutathione (100 mg/kg body weight) for the positive-control group. EPPH (0.08, 0.16, and 0.32 mL/kg body weight) was orally provided with a feeding needle for the L-EPPH, M-EPPH, and H-EPPH groups, respectively, for 6 weeks. The rats that had no exercise-induced fatigue, and oral administration of distilled water was designated as the no-exercise group. SOD, superoxide dismutase; GSH-Px, glutathione peroxidase; BUN, blood urinary nitrogen; TNF-α, tumor necrosis factor α; IL-1β, interleukin-1β; AST, aspartate aminotransferase; ALT, alanine aminotransferase. ^a,b,c,d^ Different superscript letters beside SD indicate significant differences among the groups by Tukey test at *p* < 0.05.

**Table 4:** Metabolic parameters related to exercise-induced fatigue in the liver and muscle at 30 min of intense exercise after the 6-week intervention.

	Control (*n* = 10)	Positive-Control (*n* = 10)	L-EPPH (*n* = 10)	M-EPPH (*n* = 10)	H-EPPH (*n* = 10)	No-Exercise (*n* = 10)
In gastrocnemius muscle						
Lactate (mg/mg protein)	4.34 ± 0.19 ^a^	4.03 ± 0.12 ^b^	4.33 ± 0.18 ^a^	3.73 ± 0.20 ^c^	3.57 ± 0.31 ^c^	3.88 ± 0.19 ^bc^
LDH activity (U/mg protein)	69.4 ± 3.40 ^a^	62.0 ± 3.45 ^b^	65.2 ± 4.21 ^b^	64.3 ± 1.87 ^b^	57.2 ± 3.94 ^c^	64.2 ± 4.65 ^b^
Creatine kinase (U/mg protein)	36.6 ± 0.79 ^c^	34.2 ± 0.74 ^a^	33.8 ± 0.81 ^b^	32.8 ± 1.01 ^b^	32.4 ± 3.2 ^a^	33.9 ± 0.66
Lipid peroxides (MDA nmol/mg protein)	5.42 ± 0.48 ^a^	4.57 ± 0.37 ^b^	5.37 ± 0.51 ^a^	4.62 ± 0.42 ^b^	4.17 ± 0.36 ^c^	3.96 ± 0.34 ^c^
SOD (U/mg protein)	3.66 ± 0.05 ^d^	4.25 ± 0.21 ^b^	3.92 ± 0.07 ^c^	4.51 ± 0.16 ^b^	4.91 ± 0.11 ^a^	4.08 ± 0.16 ^c^
GSH-Px (U/mg protein)	43.7 ± 1.38 ^d^	65.6 ± 1.75 ^b^	48.4 ± 1.71 ^c^	63.6 ± 1.64 ^b^	69.5 ± 2.01 ^a^	47.5 ± 1.76 ^c^
Glycogen (mg/g tissue)	7.14 ± 0.52 ^d^	8.85 ± 0.89 ^b^	7.87 ± 1.05 ^c^	9.80 ± 0.97 ^a^	9.34 ± 1.06 ^b^	10.3 ± 1.34 ^a^
In liver						
Glycogen (mg/g tissue)	0.76 ± 0.08 ^d^	0.95 ± 0.08 ^c^	0.89 ± 0.07 ^c^	1.08 ± 0.09 ^b^	1.15 ± 0.08 ^b^	1.34 ± 0.09 ^a^
Lipid peroxides (MDA nmol/ mg protein)	8.86 ± 0.78 ^a^	6.47 ± 0.71 ^b^	8.14 ± 0.77 ^a^	7.08 ± 0.65 ^b^	6.76 ± 0.69 ^bc^	6.17 ± 0.59 ^c^
SOD (U/mg protein)	2.45 ± 0.21 ^c^	3.34 ± 0.24 ^b^	2.58 ± 0.17 ^c^	3.42 ± 0.23 ^b^	4.05 ± 0.28 ^a^	3.27 ± 0.27 ^b^
GSH-Px (U/mg protein)	31.5 ± 1.08 ^d^	45.8 ± 1.34 ^b^	33.9 ± 1.31 ^c^	44.9 ± 1.54 ^b^	49.8 ± 1.38 ^a^	35.1 ± 1.34 ^c^

Values represented means ± standard deviation (SD; *n* = 10). The rats with exercise-induced fatigue had oral administration of distilled water for the control group, porcine placenta enzyme hydrolysates (EPPH) dissolved in distilled water for experimental groups, and glutathione (100 mg/kg body weight) for the positive-control group. EPPH (0.08, 0.16, and 0.32 mL/kg body weight) was orally provided with a feeding needle for the L-EPPH, M-EPPH, and H-EPPH groups, respectively, for 6 weeks. The rats that had no exercise-induced fatigue and oral administration of distilled water were designated the no-exercise group. LDH, lactate dehydrogenase; SOD, superoxide dismutase; MDA, malondialdehyde. ^a,b,c,d^ Different superscript letters beside SD indicate significant differences among the groups by Tukey test at *p* < 0.05.

**Table 5:** Metabolic parameters related to exercise-induced fatigue in the brain after the 6-week intervention.

	Control	Positive-C	L-EPPH	M-EPPH	H-EPPH	No-Exercise
Hypothalamic corticosterone expression (AU)	1 ^a^	0.88 ± 0.04 ^b^	1.05 ± 0.08 ^a^	0.66 ± 0.04 ^c^	1.71 ± 0.04 ^c^	0.71 ± 0.03 ^c^
Hypothalamic lipid peroxides (MDA nmol/mg protein)	54.5 ± 4.6 ^a^	47.2 ± 4.2 ^b^	47.4 ± 7.2 ^b^	33.2 ± 3.9 ^d^	39.9 ± 4.1 ^c^	34.8 ± 3.7 ^d^
Hippocampal TNF-α (pg/mg protein)	10.7 ± 1.03 ^a^	8.55 ± 0.97 ^b^	8.65 ± 0.97 ^b^	7.75 ± 0.65 ^c^	7.41 ± 0.51 ^c^	7.85 ± 0.73 ^c^
Hippocampal IL-1β (pg/mg protein)	6.84 ± 0.47 ^a^	5.81 ± 0.46 ^b^	5.98 ± 0.61 ^ab^	5.04 ± 0.52 ^c^	4.97 ± 0.41 ^c^	6.12 ± 0.34 ^b^
Hippocampal BDNF expression (AU)	1 ^a^	1.18 ± 0.11 ^b^	0.92 ± 0.11 ^ab^	1.45 ± 0.10 ^bc^	1.37 ± 0.09 ^bc^	1.47 ± 0.14 ^c^

Values represent means ± standard deviation (SD; *n* = 10 for protein contents and *n* = 4 for mRNA expression). The rats with exercise-induced fatigue had oral administration of distilled water for the control group, porcine placenta enzyme hydrolysates (EPPH) dissolved in distilled water for experimental groups, and glutathione (100 mg/kg body weight) for the positive-control group. EPPH (0.08, 0.16, and 0.32 mL/kg body weight) was orally provided with a feeding needle for the L-EPPH, M-EPPH, and H-EPPH groups, respectively, for 6 weeks. The rats that had no exercise-induced fatigue and oral administration of distilled water were designated the no-exercise group. TNF-α, tumor necrosis factor α; IL-1β, interleukin-1β; AU, arbitrary unit. ^a,b,c,d^ Different superscript letters beside SD indicate significant differences among the groups by Tukey test at *p* < 0.05.

## Data Availability

The data presented in this study are available on request from the corresponding author.

## Supplementary Materials

The following supporting information can be downloaded at: https://www.mdpi.com/article/10.3390/biology11121739/s1, Table S1. Primer sequences for the genes.
