# Supplementary material for: Alleviation of Cognitive and Physical Fatigue with Enzymatic Porcine Placenta Hydrolysate Intake through Reducing Oxidative Stress and Inflammation in Intensely Exercised Rats
Source: Biology (Basel). 2022 Nov 29;11(12):1739. doi: 10.3390/biology11121739 (PMC9774658; doi:10.3390/biology11121739)
Supplement: Supplementary file 1 [file biology-11-01739-s001.zip › biology-2018692-supplementary.pdf]

Table S1. Primer sequences for the genes

	Forward	Reverse
TNF-α	ACCCCCAACCTATGAAGAAA	TCCACGCAAAACGGAATGAA
IL-1β	TTGTGGCTGTGGAGAAGCTG	GCCGTCTTTCATACACAGG
BDNF	ATGCCGAACTACCCAATCGT	GCCAATTCTCTTTTTGCTATCCA
β-actin	AGCGTGGCTACAGCTTCACC	AAGTCTAGGGCAACATAGCAC AGC
